# Using Social Marketing to Manage Population Health Performance

**Published:** 2010-08-15

**Authors:** Michael L Rothschild

**Affiliations:** The Wisconsin School of Business, University of Wisconsin

## Abstract

Population health can be affected by implementing pay-for-performance measures with key players. From a social marketing perspective, people (both consumers and managers) have choices and will do what they perceive enhances their own self-interest. The bottom-up focus of social marketing begins with an understanding of the people whose behaviors are targeted. Desired behavior results when people perceive that they will get more value than the cost of behaving and when the resulting offer is perceived to be better than what is obtainable through alternative choices. Incentives should be offered to consumers; managers should receive motivation for their own behavior and understand how to motivate relevant consumers. Pay can be monetary or nonmonetary, tangible or intangible. Everyone is paid for performance. Some are paid well enough to behave as desired; others are offered a poor rate of pay and choose not to behave.

Organize policy and strategy so that self-interest does what the community requires.

Adapted from LeGrand (1)

## Introduction

This article is one in a series published by *Preventing Chronic Disease* (*PCD*) that discusses pay for performance (P4P). It considers social marketing as a well-developed managerial paradigm that can contribute to the key components of P4P as it, in turn, contributes to improving population health. Policy makers who show discomfort in engaging in P4P are not avoiding it but are merely paying poorly and allowing alternative choices a more favorable standing. From a social marketing perspective, the question driving this series of *PCD* articles is not "Should we use P4P to improve population health?" but "Should we execute P4P well or poorly?"

According to the concept of social marketing, people have choices and will act to enhance their own self-interests in the constraints of time and place. Any behavior takes place in a setting where alternative choices are available, and each is a combination of short-term and long-term costs and benefits assessed by someone with a personal (often intuitive and implicit) calculus who weighs the choices on the basis of their component features. A P4P offer is chosen if perceived as the best available deal; if not chosen, then either the "pay" was inadequate or the required "performance" was too demanding. In this article, I consider the importance of providing appropriate incentives both to managers and consumers and of assisting managers in motivating relevant consumers.

Certain terms will be used throughout this article. *People* applies to both consumers and managers, *consumers* describes people who ultimately behave to shape population health metrics, and *managers* describes people who can influence the social and physical environmental conditions that make it more or less difficult for people to behave in a certain way. Managers should be motivated to behave as desired, and, in turn, to motivate. Managers exist at many levels (eg, policy makers, manufacturers, teachers, grocers, restaurateurs, counselors). Often, disparate actors need to work together. In some cases actors will see a common P4P that benefits each, but often each actor requires a P4P offer that provides an individual benefit.

*Behavior* refers to the observable and measurable action that must occur at the individual level to establish the desired population health metric. Developing awareness and attitude are useful and often necessary, but are not sufficient. P4P may be new to population health, but the concept of appealing to self-interest in exchange for behavior is quite old and is the basis of large parts of 2 core disciplines: economics (2) and psychology (3). Public health issues such as tobacco, drug, and alcohol abuse have long built upon a base of behavior change and positive reinforcement (4,5), but the introduction of P4P into population health has been recent (6).

In commercial marketing, a consumer is offered the opportunity to "perform" an act — for example, purchasing and consuming a soft drink — and is then "paid" or rewarded with a result — in this case, refreshment and a jolt of energy from the sugar and caffeine. In public health, a person is offered the opportunity to "perform" an act — for example, wearing a seat belt — and is then "paid" or rewarded with a result — in this case, an enhanced feeling of safety. In both examples, if the person finds the P4P exchange pleasing, then he will continue to perform and to be paid.

In each case, managers also should be paid to perform. The grocer is paid to stock a soft drink and may be paid more to display it more prominently than soft drinks produced by that company's competitors. Engineers are paid to develop a seat belt that is easy to use and may be paid more if it also is comfortable to wear. Pay may be monetary or nonmonetary, such as through the esteem of one's peers for a job well done.

Population health focuses on managing distal macrolevel dependent variable metrics, such as percentage of the population that is obese. Although 90% of health determinants result from individual behavior and social and physical environmental conditions (7), 95% of health expenditures go to treatment rather than prevention (8). In the past, P4P has focused on offering financial incentives for health care organizations and personnel, but an emerging view of population health recognizes that P4P must also include rewarding managers of nonmedical components of social and physical environments (9).

Social marketing considers the same metrics, but dependent variables are more likely to be specific, proximal, microlevel behaviors, such as amount of exercise per week. Managers are rewarded for creating an environment in which exercise can more easily take place; consumers are rewarded for exercising.

Social marketing and population health are complementary. Population health has the goal of changing macrolevel societal metrics. Social marketing is silent as to the selection of metrics but provides strategic insights on how to reach the goal by considering several microlevel individual behaviors that should be changed or maintained to accumulate to a macrolevel societal change. Population health policy makers decide on resource allocations with respect to segments of the population and metrics to change. Social marketing practitioners contribute by developing efficient and effective strategies that lead to behavior changes.

## Marketing and Social Marketing

Three general tools are used to manage public health behaviors: education, enforcement, and environment (10,11). Education primarily uses messages to inform and persuade but occasionally can reinforce behavior. Enforcement uses the law to coerce, punish, or threaten to punish in exchange for appropriate behavior. The environment is used to reward desired behavior, to increase benefits, to decrease barriers for desired choices, and to decrease the hassles of daily life. Social marketing is used to manage the environment so that appropriate behavior will result. Although this simple categorical scheme can be used to provide an introduction to social marketing, reality is more ambiguous.

Marketing is "the activity, set of institutions, and processes for creating, communicating, delivering, and exchanging offerings that have value for customers, clients, partners, and society at large" (12). Social marketing is the application of commercial marketing to nonbusiness situations. The exchange is the fundamental relationship on which market systems are built. Strategies begin with a bottom-up focus that leads to an understanding of the people whose behaviors are being targeted.

In the past, much of what has been called "social marketing" in public health has not been marketing but rather has been limited to communications (13). Although many communications cases self-define as "social marketing," few cases are consistent with the previous definition. This distinction is crucial if social marketing is to contribute to P4P.

The environment can encourage exchange through the development of a choice with comparative advantage, favorable cost-benefit, and the convenience of time and place. After the choice is developed, messages are used to describe and advocate. Marketers manage through the use of the 4 *P's* (product, price, place, and promotion):

- **The** ***product*** consists of the bundle of "goods," or benefits, that a person receives in return for the desired behavior. Anything received is considered P4P and can be monetary or nonmonetary, tangible or intangible.
- **The** ***price*** consists of the bundle of "bads," or costs, that a person incurs to receive the goods. These also can be monetary or nonmonetary, tangible or intangible.
- **The** ***place*** considers the time and location for the exchange to occur. It can be a benefit or a cost, depending on its convenience.
- **The** ***promotion*** consists of the messages that announce the proposed exchange (the product, the price, the place, and the desired behavior).

The development of the package of costs and benefits must be considered in the desired behavior of both the manager and the consumer.

P4P can be seen as an example of an instrumental stimulus–response–reinforcement model. The presentation of the offer through messages is the stimulus, the desired behavior is the response, and the delivery of the package of goods and bads is the reinforcement. Social marketing gives the manager a tool kit for developing a favorable package of stimuli and reinforcers.

Other major foci that social marketing brings to P4P are an understanding of the following concepts:

- **The *person*.** Social marketing begins with a bottom-up focus to develop an understanding of people who should be motivated. Barriers that keep behavior from occurring are key and may include environmental difficulties and the hassles of daily life. Motivating benefits emerge from an understanding of the barriers and the desired behavior. After understanding barriers and benefits, descriptors can be developed on the basis of, for example, demographics, psychographics, and geographics.
- **The *segment*.** Marketers divide people into groups with similar needs, motivations, barriers, or behaviors, with the goal of maximizing pursuit of the population metric. A segment may be a group that is easiest to target or one that is disadvantaged in some way.
- **The *competition*.** Whenever there is free choice there is competition, yet too often this is ignored by managers. Broccoli or a jelly doughnut. Safe or risky sex. Binge or moderate drinking. Without understanding the alternative choices and their appeal, the offer may be too weak to be accepted.
- **The *position*.** The offer must be developed so that it is perceived as the most desirable choice possible at the moment of decision making.
- **The *exchange*.** An offer of P4P is made.

These points appear to focus on consumers, but they are equally valid for managers who need to overcome barriers in their own hassled lives, who work with insufficient resources, who make decisions from a set of competing alternative opportunities, and who realize a positive outcome for their own careers and organizations.

## A Social Marketing View of "Pay"

In the stimulus–response–reinforcement model, pay is the reinforcement. Social marketing considers pay in several ways:

- **Monetary and nonmonetary, tangible and intangible benefits and costs.** Employees may receive the financial benefit of reduced insurance payments if they join a workplace wellness program, but they also may receive recognition for achieving weight loss, social support for joining a walking club, or the ability to more easily play active games with their children. Costs can also be monetary or nonmonetary. These include time (it takes too long to work out), hassle (it takes 2 buses in each direction to use the gym), or ego (embarrassment at showing one's overweight body).
- **Cost–benefit relationship.** Often people do not behave as desired because they are unwilling to do so. The perceived bundle of benefits must exceed the perceived bundle of costs. Pay is the benefit relative to the cost and cannot be considered in isolation.
- **Competitive alternative choices.** In a free-choice society, the cost–benefit package must be perceived to be more favorable than all alternative choices.
- **Short-term versus long-term costs and benefits of all choices.** Although policy makers may consider long-term good health to be the ultimate pay, consumers and managers often are short-term maximizers. In a simple world there may be only 2 choices: good and bad. "Good" choices, such as exercising and eating healthfully, have short-term costs (eg, learn to cook, recover from painful exercise), and the eventual benefits of good health are large, distant, and not guaranteed. "Bad" choices such as playing video games and eating pizza have short-term benefits (eg, it is fun, it tastes good), and the eventual costs of poor health are large, distant, and not guaranteed. Inspiring people to engage in behavior with long-term benefits or short-term costs when competitive offerings promise instant gratification is difficult. The "tyranny of small decisions" (14) explains that there are many opportunities during the day for immediate gratification (fast-food breakfast, 10:00 am doughnut, evening video game with ice cream), and these often keep people from moving toward their long-term goal of good health. P4P should consider immediate and future pay relative to the cost–benefit of the desired and the competitive choices.

## A Social Marketing View of "Performance"

In the stimulus–response–reinforcement model, performance is the response. Stages-of-change models have long been suggested in both marketing and public health strategies (15), and managers typically express their performance goals relative to these dependent variable responses. Marketing managers understand that behavior is what ultimately must change. Therefore, to contribute to changing population health metrics, P4P must focus on behavior.

Performance requires an examination of the barriers to behavior. Considering benefits without first understanding barriers can result in a weaker stimulus for change.

Barriers must be overcome before benefits are offered. Often people do not behave as desired because they are unable to do so. A consumer may desire the benefits offered by an employer's wellness plan but may not be able to move toward behavior change until the barriers (eg, lack of ability to cook, lack of proper exercise attire, fear of injury from exercising, an already overburdened and hassled life) are reduced. Once barriers are reduced, cost–benefit can be considered.

A potential failing of P4P can be misunderstanding the desired performance. An example of this is the use of P4P in health care cases when the terms of the exchange were not properly stated. Some medical facilities and physicians may have performed to maximize number of patients seen or to maximize pay on a per capita basis, rather than to maximize wellness of patients. Prospective exchange partners will interpret the offering through their own lens of self-interest. P4P can be a powerful tool, but it is expensive and must be used with great care.

## Who Needs to Receive P4P?

In considering P4P, marketers target 2 types of people in terms of population health metrics: consumers, already discussed extensively, and managers.

Managers are the stewards of social and physical environments but also are people who respond or resist. They too exist in a world of barriers, insufficient benefits, continual hassles, and strong competitive pulls on scarce financial and time resources. At each level of management there is a person who needs to be motivated to behave and who also needs to motivate other people. The 4 P's are relevant for both the consumer and the manager.

A manager's self-interest is driven by both the needs of the organization and personal needs. Organizations provide incentives for their managers through pay, performance incentives, personnel reviews, promotions, and the esteem of cohorts and more senior members of the organization. If these incentives are properly crafted, managers will behave in their own self-interest to further the greater interests of the organization. The organization acts in its own self-interest by motivating its managers to achieve the organization's goals.

For example, until recently most firms did not see the benefit to the firm of providing wellness programs for employees. "We tried to get firms to adopt wellness because it was the right thing to do, but that failed. Now we show them how it reduces costs and increases profit, and that works" (16). P4P has been demonstrated to show savings of more than $5.50 in medical costs and reduced absenteeism for each dollar invested in workplace wellness programs (17). Employers are managers who need to be motivated and also should motivate others.

Three major segments of both consumers and managers exist:

- Those who are prone to behave appropriately, and are able to do so, may need only messages to remind them.
- Those who are resistant may need the force of law as motivation (11).
- Those who are aware and motivated but who are unable to behave may be the segment most likely to respond to P4P. Reducing barriers and increasing benefits among those who are unable or unwilling may provide sufficient environmental change to allow behavior to occur.

## Some Concluding Thoughts

In 2000, the Wisconsin Department of Transportation chose alcohol-related crashes as a metric of concern. After extensive research, talking to the target of single men aged 21 to 34 years who drove while impaired, Road Crew emerged as a fee-based ride program in rural communities. The program provided consumers limousine rides to, between, and home from taverns so that they would leave their vehicles at home. In the past, men were not able to admit to their friends that they were too drunk to drive, but now they could be seen as "cool" because they used the limousine. In P4P terms, men were paid with an evening of rides in a limousine in return for the performance of not driving.

This program to change proximal behavior was measurable. It was aimed at the population segment most likely to have a motor-vehicle accident while impaired, offered a favorable exchange, and gave more than 85,000 rides in 6 communities over 5 years. It prevented approximately 140 motor-vehicle accidents, reduced motor-vehicle accidents by 17% in relevant communities, saved the citizens of the state approximately $30 million, and was financially self-sustainable (primarily from ride fees). The population health top-down perspective determined the macrolevel goals, while the social marketing bottom-up perspective led to an understanding of the people whose behavior needed to change and the environmental changes that were needed to facilitate the behavior change (18).

Population health performance metrics can be achieved through paying for specific performances. Programs can be developed through the use of social marketing and its 4 P's. From a social marketing perspective, for P4P to succeed it must accommodate self-interest. The pay may be monetary or nonmonetary but must exceed the cost of the behavior, be better than that offered by alternative choices, and show a short-term as well as long-term benefit.

Much public health work has focused on telling people what to do, under the assumption that if people knew what to do, they surely would change their behaviors to do what is "right." This has led to less than ideal results, and, in turn, a call for P4P. Perhaps an adoption of the social marketing paradigm can lead to a greater effect from P4P and to more population health successes. Every choice has costs, benefits, and competitive options. It is the task of policy makers to establish the terms of P4P so that they are likely to be accepted by consumers and managers.
